# Prevalence and Genotype Distribution of Human Papillomavirus in Invasive Cervical Cancer, Cervical Intraepithelial Neoplasia, and Asymptomatic Women in Southeast China

**DOI:** 10.1155/2018/2897937

**Published:** 2018-10-08

**Authors:** Xuelian Wang, Yanli Zeng, Xiumin Huang, Youzhong Zhang

**Affiliations:** ^1^Department of Gynecology and Obstetrics, Qilu Hospital of Shandong University, Jinan 250012, China; ^2^Department of Gynecology and Obstetrics, Zhongshan Hospital of Xiamen University, Xiamen 361004, China; ^3^Center of Clinical Laboratory, Zhongshan Hospital of Xiamen University, Xiamen 361004, China

## Abstract

Cervical cancer is one of the leading causes of cancer-related deaths among women and it is caused by the human papillomavirus (HPV). High variation has been reported in the attribution of specific HPV genotypes to cervical neoplasia among various geographic regions. For effective control of cervical cancer through HPV vaccination, it is essential to estimate the cost-effectiveness of vaccination, to monitor the potential transition into other HPV genotypes, and to understand the distribution of specific HPV genotypes across a specific geographic region. In this study, the distribution of HPV genotypes was investigated in southeast China, from 2011 to 2016. The 12,816 cervical swabs collected from women (age 18–78 years, median 43.6 years) outpatients were analyzed. HPV prevalence among 12,816 cervical swabs analyzed was 22.3% (2,856/12,816). Among these positive cases, 2,216 had only one HPV genotype while 640 had multiple HPV genotypes. The cases with multiple types revealed 23 different HPV genotypes with the five most prevalent being HPV18 (18.2%), HPV52 (14.1%), HPV16 (11.9%), HPV58 (10.6%), and HPV33 (5.5%). The rates of HPV infection in patients with cervical inflammation, CIN-1, CIN-2, CIN-3, squamous carcinoma, and adenocarcinoma were 38.4%, 80.5%, 82.6%, 92.3%, 97.5%, and 93.4%, respectively. Four HPV genotypes, HPV18, HPV16, HPV52, and HPV58, were more prevalent in patients with CIN-2-CIN-3 and invasive cervical cancer. A comparison of HPV genotypes attribution to cervical cancer between southeast China and global incidences revealed distinct differences. Due to this unique prevalence, it is essential to streamline the vaccination development protocol prior to administering vaccines based on global data.

## 1. Introduction

Cervical cancer is one of the most prevalent cancers among women [1] with 527,600 new cervical cancer cases diagnosed globally and 265,700 cervical cancer-related deaths just in 2012 [2]. In China, cervical cancer is prevalent among women aged 15–44 years and is the eighth most common cancer affecting women [3]. The incidence of cervical cancer in China was 62 per 100,000 and about 50% among them resulted in deaths (30 per 100,000) in 2012 [2]. Epidemiological evaluations estimated that, without intervention, the occurrence of new cervical cancers in China could increase 40–50% from 2010 to 2050 [4].

Human papillomavirus (HPV) infection, the most frequently diagnosed sexually transmitted infection, has been determined to be the primary cause of cervical squamous intraepithelial lesions and invasive cervical cancer. HPV is a double stranded DNA virus belonging to the genus* Papilloma* in the Papovaviridae family [5] with more than 200 known HPV genotypes. HPV has a preference to infect human mucosa and skin, and at least 40 HPV genotypes infect the genital tract mucosa. Among these, 15 (HPV16, 18, 31, 33, 35, 39, 45, 51, 52, 56, 58, 59, 68, 73, and 82) are classified as high-risk for the development of cervical cancer [6, 7].

A proactive measure to reduce the incidence of HPV infection is vaccinating adolescent women and men against the virus before the first sexual encounter. There are two prophylactic vaccines (Cervarix™ by GlaxoSmithKline, United Kingdom, and Gardasil™ by Merck Sharp and Dohme, NJ, USA) that are presently available for the prevention of HPV infection and the consequent progression of cervical dysplasia. These vaccines are effective against the HPV16 and HPV18 genotypes, which account to about 70–80% of global invasive cervical cancers [8–11]. However, the vaccines have not been effective against non-16/18 HPV genotypes due to their unpredictable distribution in various geographical regions [12–15]. To address this issue, it is essential to perform a population-specific assessment of the attribution of each HPV genotype to the prevalence of cervical cancer. Such parameters are essential to evaluate the vaccination benefits and cost-effectiveness of current and next generation vaccines and formulate an HPV-based screening strategy. These factors will help curtail the spread of HPV types in southeastern China.

Current reports estimate that 85,000 new cases of HPV-related cancer and 75,000 cases of cervical cancer are diagnosed per year in China [16]. In previous studies, HPV 16, HPV 58, and HPV 33 were the most prevalent HPV types in Beijing [17]; by far the most commonly found type was HPV16, followed by HPV 58, 52, 33, and 18 in Shanxi Province [18]; HPV 16, 52, and 58 were found the most frequently genotypes in Liaoning Province [19]; the most common subtype was HPV16, and other common high-risk types included HPV58 and 39 in Xinjiang [20], HPV16 being the most common, followed by HPV52, 58, 6, and 53 in Qingdao City [21]; HPV 16 and 18 are the most common types of infection among cervical cancer patients, followed by HPV 58, 56, and 59, which is different from the high HPV 58 infection rate of outpatients in South West China [22]. To effectively use the HPV vaccines and mitigate cervical cancer in China, it is essential to understand the prevalence of HPV and its type distribution in specific geographical regions, which are not clearly known. Therefore, in this study, we determined distribution of HPV genotypes and the correlation between cervical lesions and HPV infections among women in southeast China, which has 69/100,000 cervical cancer incidences among young women. The discoveries of this evaluation may offer new insight for immunizations to efficiently prevent cervical cancer in this area.

## 2. Materials and Methods

### 2.1. Study Population and Sample Collection

In this study, we analyzed 12,816 cervical swabs collected from women (age 18–78 years, median 43.6 years) outpatients who came for gynecological examination to the Department of Obstetrics and Gynecology of Xiamen Zhongshan Hospital (Xiamen City), Fujian Provincial Hospital (Fuzhou City), and Zhejiang Hospital (Hangzhou City), between January 2013 and June 2016. The Xiamen Zhongshan Hospital is a tertiary institution with 2,500 beds and is affiliated with the Xiamen University. Fujian Provincial Hospital is a tertiary institution with 2,300 beds. It is affiliated with the Fujian Provincial Health and Family Planning Commission. Zhejiang Hospital is also a tertiary institution. It has 2,200 beds and is affiliated to the Zhejiang Provincial Health and Family Planning Commission. A woman was included if she had an active sexual history, was a gynecological outpatient, and had symptoms of genital tract diseases, including cervicitis and vulvar irritation, had never had a miscarriage or was not pregnant, had not had a complete uterus or cervix resection, and consented to an HPV test and cervical cytology assessment. Specimens were just obtained from the women who consented to an HPV test and cervical cytological assessment. Techniques performed in this evaluation were authorized by the Research Ethics Boards at the School of Public Health, Xiamen University.

A gynecologist collected the following samples from each woman using routine hospital protocols. Exfoliated cell samples were obtained from the cervix. Cell scrapings were collected from the cervix by using a cytobrush from the ecto- and endocervix of the uterus. The cell samples on the cytobrush were suspended in a specimen transport medium (Yaneng Biotechnology Limited Corp., Shenzhen, China) and stored at −70°C until DNA extraction. Identification of HPV and pathology diagnosis were performed independently. All abnormal slides showing likely pathology were visualized by two independent surgical pathologists.

### 2.2. DNA Extraction and HPV Genotyping

DNA was separated from cervical exfoliated cell specimens kept in specimen transport medium and purified based on the company's directions (Genomic DNA Kit, Yaneng Biotech, Shanghai). HPV was detected by amplifying a 450-bp region in the L1 gene by polymerase chain reaction (PCR) using the primer pair PGMY09/11 as described previously [18]. HPV-positive samples were typed by the Linear Array HPV Genotyping Test (Roche, Molecular Systems, Inc., CA) according to the manufacturer's instructions. This assay can distinguish the HPV genotypes 6, 11, 16, 18, 26, 31, 33, 34 (formerly 64), 35, 39, 40, 42, 45, 51, 52, 53, 54, 55 (formerly 44), 56, 58, 59, 61, 62, 64, 66, 67, 68, 69, 70,71, 72, 73, 81, 82 (formerly IS39 or MM4), 83, 84 (formerly MM8), and 89 (formerly CP6108). In short, PCR was conducted in a 100-*μ*l reaction mixture with 50 *μ*l of DNA, 50 *μ*l of master mix containing Tris buffer, KCl, MgCl_2_, AmpliTaq Gold DNA polymerase, dNTPs, and biotinylated PGMY09/11 primers. The PCR cycling conditions were incubation at 50°C for 2 min then 95°C for 9 min, followed by 40 cycles of 30 sec at 95°C, 1 min at 55°C, and 1 min at 72°C. A final extension was performed at 72°C for 5 min. The amplified products were purified, denatured, and hybridized with the biotinylated HPV-genotype-specific probes, which were blotted onto genotyping strips.

Each sample quality was verified by PCR amplifying of the beta globulin gene using the primers Beta-GP1/2 as described previously [23]. CaSki and HeLa cell preparations were used as positive controls in PCR. Both cell preparations were diluted such that they contain approximately 50 copies of HPV16 and HPV18, respectively. A no DNA negative control was included per five samples.

### 2.3. Cytology and Histopathology

Specimens were shipped to a central laboratory in the School of Public Health, Xiamen University (SPHXM), for processing and testing. Cervical samples were evaluated using the* ThinPrep* Pap Test (Hologic Inc., Bedford, MA) and results were recorded according to the Bethesda 2001 classification system [24]. The following terminologies were used in the reports: atypical squamous cells of undetermined significance (ASC-US), atypical squamous cells-cannot exclude HSIL (ASC-H), low-grade squamous intraepithelial lesions (LSIL), high-grade squamous intraepithelial lesions (HSIL), and squamous cell carcinoma (CC).

Patients showing abnormal cytology underwent an additional colposcopic biopsy. For visible lesions, direct biopsy was conducted. For patients without visible lesions, four tissue samples were collected from the 3, 6, 9, and 12 o'clock positions in the conjunction belt of the cervix. Histopathological examination was performed by a panel of expert gynecological pathologists at Xiamen Zhongshan Hospital. Final results of patients with cervical intraepithelial neoplasia (CIN) were determined by an independent endpoint committee after reviewing all data. Based on the review, patients were classified as normal, CIN-1, CIN-2, CIN-3, cervical cancer, or adenocarcinoma.

### 2.4. Statistical Analyses

Descriptive statistics were used to define clinical data. For each HPV genotype, the crude overall prevalence rate was expressed as the percentage of the value obtained by dividing the total number of positive specimens (encompassing the single-type and multiple-type infections) by the total number of adequate specimens examined for the disease category. The relative positive rate or relative distribution used the HPV-positive sample number as the denominator. The attribution of HPV genotypes in multiple-type infections was estimated using a previously published method [25, 26]. Briefly, the attribution factor of each genotype was calculated by adding the “crude prevalence of single-type infection” plus “crude prevalence of multiple-type infections.” The risk was estimated by calculating the odds ratio (OR) and 95% confidence interval (CI), and reference group for calculating of OR is that the majority of women outpatients had no cytological abnormalities. Data were analyzed using IBM SPSS Statistics version 20 software (IBM Corp., Armonk, NY) and *P-*values < 0.05 were considered significant.

## 3. Results

### 3.1. HPV Prevalence and Genotyping in Women Outpatients

**Figure 1:**
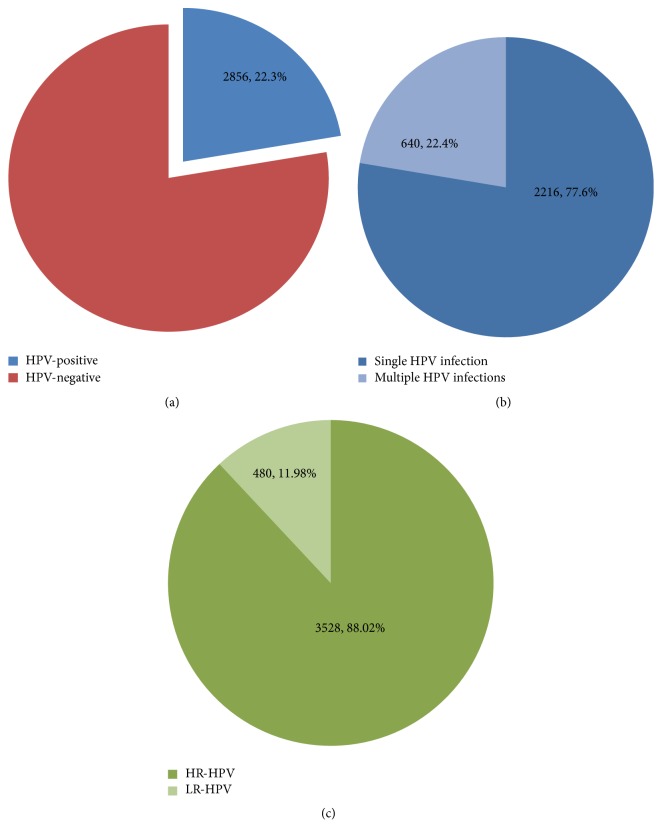
HPV pervasiveness. (a) Pervasiveness of HPV-positive specimens. (b) Single and multiple HPV infections. (c) Frequency of high-risk and low-risk HPV types of HPV-positive specimens. The total frequency of 4,008 surpassed the number of positive samples (2,856) since every single genotype was counted independently in multiple infections.

During the 3-year study, a total of 22.3% (2,856) of the 12,816 women outpatients tested positive for one or more of the HPV genotypes (Figure 1(a)). Among them, 77.6% (2,216/2856) tested positive for single HPV infection and 22.4% (640 /2856) for multiple HPV infections (Figure 1(b)). In addition, of the 2,856 total positive specimens, the numbers of high-risk HPV (HR-HPV) genotypes and low-risk HPV (LR-HPV) genotypes were 3,528 and 480, respectively (Figure 1(c)). The total frequency, calculated by considering each infection in the multiple infection samples separately, accounted to 4,008 thereby exceeding the number of positive samples (2,856).

**Figure 2:**
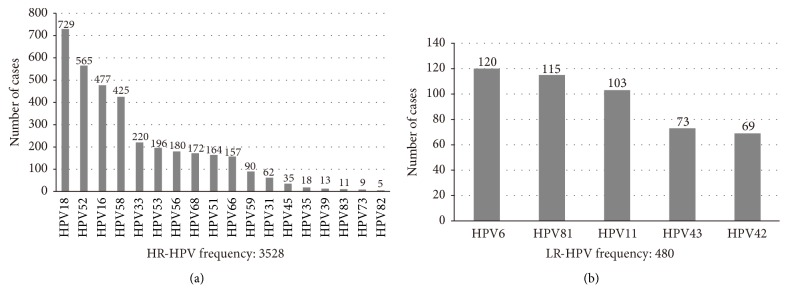
Distribution of HPV genotypes of the HPV-positive specimens. (a) High-risk HPV genotypes. (b) Low-risk genotypes.

**Figure 3:**
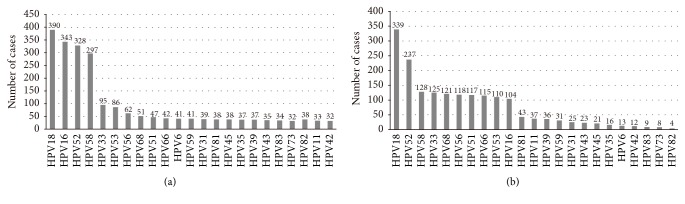
Distribution of HPV genotypes in single (a) and multiple (b) HPV infections.

As revealed in Figure 2(a), the top ten HR-HPV genotypes are HPV18 (729,18.2%), followed by HPV52 (565,14.1%), HPV16 (477,11.9%), HPV58 (425,10.6%), HPV33(220,5.5%), HPV53(196,4.9%), HPV56 (180,4.5%), HPV68 (172,4.3%), HPV51 (164,4.1%), and HPV66 (157,3.9%). Of the LR-HPV genotypes (Figure 2(b)), HPV6 was the most common (120, 3.0%), accompanied by HPV81 (115, 2.9%), HPV11 (103, 2.6%), HPV43 (73, 1.8%), and HPV42 (69, 1.7%). In specimens with a single HPV infection, pervasiveness of the HPV types was (top ten) HPV18 (390,17.6%), HPV16 (343,15.5%), HPV52 (328,14.8%), HPV58 (297,13.4%), HPV33 (95,4.3%), HPV53 (86,3.9%), HPV56 (62,2.8%), HPV68 (51,2.3%), HPV51 (47,2.1%), and HPV66 (42,1.9%) (Figure 3(a)). In samples with multiple HPV infections (Figure 3(b)), prevalence of the top ten genotypes was as follows: HPV18 (339,52.9%), HPV52 (237,37.0%), HPV58 (128,20.0%), HPV33 (125,19.5%), HPV68 (121,18.9%), HPV56 (118,18.4%), HPV51 (117,18.3%), HPV66 (115,18.0%), HPV53 (110,17.2%), and HPV16 (104,16.2%).

### 3.2. HPV Prevalence in Patients with Different Cytological Results

The majority of women outpatients (9848/12816, 76.84%) had no cytological abnormalities at the beginning of the study. Only 23.16% (2968/12816) had cytological abnormalities, including 3.41% (437/12816) with ASC-US, 5.55% (771/12816) with ASC-H, 1.51% (194/12816) with LSIL, 7.51% (963/12816) with HSIL, and 5.17% (663/12816) with CC (Table S1). The frequency of women who tested positive for HPV by PCR increased with the increasing severity of cytological abnormalities: 5.8% (574/9848) in women with normal cytology, 50.1% (219/437) in ASC-US, 61.0% (434/711) in ASC-H, 86.0% (167/194) in LSIL, 82.9% (799/963) in HSIL, and 100% (663/663) in CC.

### 3.3. HPV Prevalence in Women with Different Pathological Diagnosis

In the 2968 women who tested positive in cytology, colposcopic biopsy was performed. A total of 22.03% (654/2968) were diagnosed with inflammation (normal), 17.79% (528/2968) with CIN-1, 21.05% (625/2968) with CIN-2, 24.05% (714/2968) with CIN-3, 10.98% (326/2968) with squamous cell carcinoma, and 4.08% (121/2968) with adenocarcinoma.

The HPV-positive rates and the quantity of multiple-type infections for every cervical pathology status are revealed in Table 1. The rates of HPV infection in patients with cervical inflammation, CIN-1, CIN-2, CIN-3, squamous carcinoma, and adenocarcinoma were 38.4%, 80.5%, 82.6%, 92.3%, 97.5%, and 93.4%, respectively. The HPV-positive rate in typical women was significantly less than CIN-1 (*p* < 0.01), CIN-2 (*p* < 0.01), CIN-3 (*p* < 0.01), and invasive cervical cancers (*p* < 0.001). The HPV-positive rates for CIN-1 and CIN-2 were significantly less than CIN-3 (*p* < 0.01) and invasive cervical cancers (*p* < 0.001). The HPV-positive rate for adenocarcinoma was less than squamous cell carcinoma (*p* = 0.01). The amount of multiple-type infections located in CIN-1 (29.0%) and CIN-2 (27.3%) lesions was significantly greater than in CIN-3 (19.2%), squamous cell carcinoma (19.6%), and adenocarcinoma (18.1%). No differences were determined in the amount of multiple-type infections among CIN-3, squamous cell carcinoma, and adenocarcinoma.

**Figure 4:**
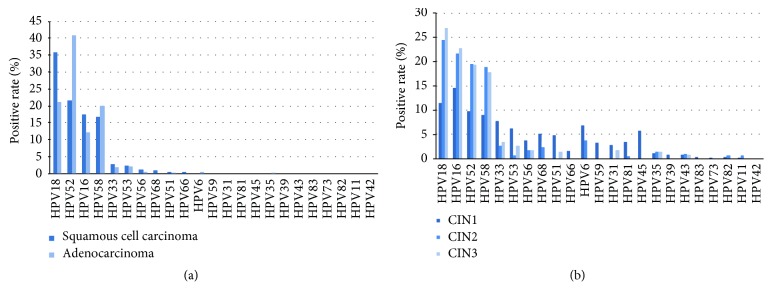
Relative distribution of HPV types of HPV-positive invasive cervical cancer and cervical intraepithelial neoplasia. (a) Relative distribution of HPV types of HPV-positive cervical squamous cell carcinoma (N = 326) and adenocarcinoma (N = 121). (b) Relative distribution of HPV types of HPV-positive cervical intraepithelial neoplasia (CIN) grade 1 (N =528), CIN-2 (N= 625), and CIN-3 (N =714). Patients with several identified HPV types are in each type and thus were counted more than once.

The relative distribution of HPV genotypes, no matter the status of single- or multiple-type infections, of the 326 HPV-positive squamous cell carcinoma specimens is revealed in Figure 4(a). HPV18 was the most frequently identified type in 35.8% of the positive specimens, accompanied by HPV52, HPV16, and HPV58 with close rates (21.6%, 17.4%, and 16.9%, respectively), and another four HPV genotypes (HPV33, 53, 56, and 68) with rates spanning from 2.7% to 0.9%. Nevertheless, HPV52 was the most frequently identified type of the 121 HPV-positive adenocarcinoma specimens with an overall (single and multiple infections combined) relative positive rate of 40.9% accompanied closely by HPV18 (21.1%). The two HPV types (HPV58 and HPV16) that followed were determined in much lower rates (20.1% and 12.3%) (Figure 4). HPV33/53/56/51/6 genotypes were rarely identified (0.3–2.0%). HPV18, HPV16, HPV52, and HPV58 were the four genotypes most frequently identified in CIN-3 and CIN-2 patients (Figure 4(b)). In comparison, the distribution was substantially altered for CIN-1, in which HPV16 was the most common type identified with an overall relative positive rate of 14.6%, accompanied by HPV18 (11.5%), HPV52 (9.7%), and HPV58 (9.0%). A broader range of nine HPV genotypes was noted in the CIN-1 positive patients (HPV16, 18, 52, 58, 33, 53, 68, 6, and 45) demonstrating an overall relative positive rate of >5%, in contrast to the four types for CIN-2 and CIN-3, respectively.

### 3.4. Relative Risk of Each HPV Genotype for Cervical Lesions

The crude OR was calculated for all 2968 patients with single and multiple HPV infections. For squamous cell carcinoma, statistically significant risk estimates were observed for HPV16, 18, 52, 58, 33, 53, and 68, with magnitudes between OR 4.70 (95% CI = 1.81-15.27) for HPV56, OR 5.74 (95% CI =2.34-12.43) for HPV31, and OR 2.61 (95% CI = 0.97-11.20) for HPV66 (Table 2). For adenocarcinoma, statistically significant risk estimates were observed for HPV16, 18, 52, 58, 33, 53, and 68, with magnitudes between OR 5.16 (95% CI = 2.42-13.75) for HPV56, OR 5.97 (95% CI = 1.14-11.87) for HPV31, and OR 2.09 (95% CI = 0.84-10.30) for HPV66 (Table 2). In patients who tested positive for CIN-1, OR was significantly higher for HPV16, 18, 52, and 58, with magnitudes between OR 5.53-10.41 for HPV31, 3353, 68, 56, 68, and 66 (Table 2). For CIN-2/3, statistically significant correlations with risk were detected for HPV16, 18, 52, and 58 (Table 2). From these findings, we concluded that HPV16, 18, 52, and 58 were the most high-risk and malignant types.

### 3.5. Age-Specific Pervasiveness of HPV

Patients were divided into six groups based on their ages (< 20 years, 21–30 years, 31–40 years, 41–50 years, 51–60 years, and > 60 years) and the existence of HPV infections in every age category was established. As demonstrated in Table 3, age-specific pervasiveness of HPV showed one peak at the youngest age category (48/145, 33.1%). The HPV-positive rates diminished slowly with age increase (Table 3).

## 4. Discussion

The pervasiveness of cervical cancer in women is rising across the globe, especially in China. Since HPV infection is the primary cause of cervical cancer [27], it is necessary to evaluate the distribution of HPV genotypes in women from the same region to examine the risk of cervical cancer. This type of data is pivotal since the distribution and pervasiveness of HPV genotypes differ by geographic areas. A large-scale evaluation was conducted to establish the distribution of HPV genotypes in women without symptoms, cervical intraepithelial neoplasia, and invasive cancers in southeastern China. During this study period, HPV vaccination rate was low in China due to the lack in government assistance to reduce vaccine cost. Hence, this data can be used as a baseline to compare HPV prevalence after rigorous implementation of HPV vaccines. This data will also aid immensely in the identification of potential HPV genotype replacement or new genotypes that may occur in future.

Details regarding the age-specific pervasiveness of HPV infection among women are pivotal for the production of vaccines and protective approaches for the prevention of cervical cancer. In the present study, HPV prevalence largely peaked in the youngest age assemblage, and the number of positive HPV cases declined progressively with increasing age. Additionally, HPV infection prevalence conformed to a bimodal U-shaped curve, which corroborates a typical observation previously reported [28], with an initial predominant peak in the <20 years' group and a subsequent smaller peak in the >60 years' group. A justification for why the initial peak occurred in younger women could be related to inadequate sex education and risky sexual conduct. Early sex education is therefore fundamental for the control of HPV infection. The appearance of the second smaller peak in more senior women may be associated with the recurrence of latent HPV infections or changes in hormones and immunity. This finding corroborates a previous observation [29] and, according to global statistics, is also a characteristic pattern in Asian women [30]. Similar papers have recently also demonstrated that HPV infection prevalence reaches its highest point in Chinese women between 20 and 24 years, thereafter decreasing considerably and then steadying by middle age [31]. In contrast to women from developed regions, the second peak was detected in women aged 45–55 years in less developed regions [31].

We found that the overall prevalence of HPV, including high-risk and low-risk HPV, among women outpatients who visited the Xiamen Zhongshan Hospital, Fujian Provincial Hospital, and Zhejiang Hospital for gynecological examination was 22.3%. This is consistent with that reported for the city of Fuzhou (22.5%) [32] but is lower than that reported for Qingdao (32.2%) [21] and Harbin (36.5%) [33]. Since the women who participated in this study were outpatients, this rate can be expected to vary in nonpatients who were enrolled in the study. Consistent with this notion, a recent multicenter, population-based cross-sectional study conducted on the entire population reported a 14.3% HPV-positive rate [29]. The authors found a unique HPV genotype distribution with HPV18 being more prevalent in this region, followed by HPV52, HPV16, and HPV58. However, a similar genotype distribution was reported in a hospital-based study in Fuzhou [32]. Others have reported the high prevalence of HPV18 and HPV52 in Hong Kong [34, 35], Macao [36], and Taiwan [37]. Various other genotype distributions have been reported for other parts of China [17–21, 31, 33, 35, 38–42] and around the world [43–46].

In this study, histological analysis served as a good indicator of HPV prevalence; i.e., the severity of the cervical lesion correlated with the presence of HPV. The frequency of HPV was 38.4% in patients diagnosed as normal, but 80.5%, 82.6%, 92.3%, 97.5%, and 93.4% in patients with CIN-1, CIN-2, CIN-3, squamous cell carcinoma, and adenocarcinoma, respectively. Among the HPV genotypes, HPV18, 16, 52, and 58 were the most prevalent in cervical intraepithelial neoplasia and invasive cervical cancers. Similarly, a higher attribution of HPV52 and HPV58 to cervical intraepithelial neoplasia and invasive cancers was reported in Hong Kong [35]. This study together with others showed that HPV52 and HPV58 are responsible for invasive cervical cancers and that they ranked among the top three HPV genotypes that cause CIN-1, CIN-2, and CIN-3 lesions. Furthermore, OR analysis revealed that, together with HPV18 and 16, both 52 and 58 may be potent oncogenic inducers. This indicates the likelihood of precancerous lesions induced by HPV18, 16, 52, and 58 infections developing into invasive cancer in comparison with other HPV genotypes that may not induce invasive cancer. Therefore, monitoring for HPV18, 16, 52, and 58 may be critical in areas where cervical cancer incidences are on the rise. In this study, we found that both HPV52 and HPV58 contributed significantly to squamous cell carcinoma. This finding is also consistent with the results reported in a previous study in East Asia [22, 30, 47]. Our evaluation additionally revealed the ascription of HPV52 and HPV58 in regard to other HPV genotypes in inducing intraepithelial neoplasia, for which information is scarce. One can assume that the administration of HPV16/18 prophylactic immunizations may alter the distribution of HPV types at minimum in patients with intraepithelial neoplasia and would act as an indicator of HPV type alteration.

Contemporary HPV vaccines aim at HPV6, 11, 16, and 18 genotypes and thus are efficient against cervical intraepithelial neoplasia, cervical cancer, and genital warts.

However, these vaccines may not effectively mitigate cervical cancer in regions such as southeast China where HPV52 and HPV58 were more prevalent.

Thus, this evaluation shows the obvious requirement to comprehend the distribution of particular HPV genotypes in a specific area before creating protective approaches.

In 2014, a nonavalent vaccine was licensed by the US Food and Drug Administration [48]. Merck used the most direct approach of all the protein-based strategies by increasing the quantity of virus-like particle (VLP) types from four to nine in their vaccine. Their nonavalent vaccine comprises VLPs of five further oncogenic varieties (HPV 31, 33, 45, 52, and 58) in addition to HPV 6, 11, 16, and 18 [48, 49]. These additional types might improve type-specific defense against approximately 70% to 90% of HPV infections that result in cervical cancers. Thus, this nonavalent vaccine might eventually effectively mitigate cervical cancer in southeast China.

In conclusion, the distribution of HPV genotype and prevalence in women outpatients in southeast China was unique with higher frequencies of HPV52 and HPV58 that may induce the development of cervical cancer from lesions. However, HPV16 and HPV18 were the more common types among patients with cervical intraepithelial neoplasia and invasive cervical cancers. Therefore, we consider that, in this region, HPV16, 18, 52, and 58 are more potent carcinogens, and women with these genotypes may be at high risk to develop cervical cancer. These discoveries enable us to suggest that the next generation HPV preventative immunizations should incorporate HPV52 and 58 genotypes to more efficiently protect women in this area of China and other areas in which similar HPV genotypes are pervasive. This evaluation also offers the basis to identify efficient clinical management approaches to avert cervical cancer in this area.

## Figures and Tables

**Table 1:** HPV positive rate and proportion of multiple-type infection according to cervical pathology status.

**Cervical Pathology status (total no. examined)**	**HPV positive rate (%)**	**Single-type infection** ^1^ ** (%)**	**Multiple-type infection** ^1^ ** (%)**		
			**2HPV types **	**3HPV types **	**4 or more HPV type**
**Normal (654) **	251 (38.4%)	77 (11.7%)	81 (12.4%)	68 (10.4%)	25 (3.9%)

**Cervical intraepithelial neoplasia (CIN)**					

**CIN1 (528)**	425 (80.5%)	272 (51.5%)	95 (17.9%)	46 (8.7%)	12 (2.4%)

**CIN2 (625)**	516 (82.6%)	346 (55.3%)	105 (16.8%)	41 (6.6%)	24 (3.9%)

**CIN3 (714)**	659 (92.3%)	522 (73.1%)	111 (15.5%)	26 (3.7%)	0

**Invasive cervical cancer (ICC)**					

**Squamous cell carcinoma (326)**	318 (97.5%)	254 (77.9%)	47 (14.5%)	14 (4.3%)	3 (0.8%)

**Adenocarcinoma (121)**	113 (93.4%)	91 (75.3%)	18 (14.8)	4 (3.3%)	0

^1^All study samples are included as denominator.

**Table 2:** The estimated risk of each HPV type for cervical lesions.

HPV types	SCC		ADCA		CIN1		CIN2		CIN3	
	OR	95%CI	OR	95%CI	OR	95%CI	OR	95%CI	OR	95%CI
HPV18	54.61	37.17–79.66	60.11	48.74–86.34	17.53	10.94–34.67	25.50	18.36–41.15	38.72	26.31–50.13
HPV16	29.05	20.84–48.72	27.09	18.91–41.31	12.19	9.51–38.60	18.62	11.34–42.03	24.34	15.43–47.31
HPV52	49.34	36.61–72.41	46.39	30.00–58.79	14.33	8.67–34.11	19.19	10.56–47.25	27.42	13.64–46.18
HPV58	35.54	18.24–53.14	37.41	21.32–47.39	16.60	8.34–37.41	21.44	13.54–41.28	30.19	13.51–60.12
HPV33	12.34	8.37–35.39	14.10	7.56–32.13	8.71	2.34–21.33	9.53	2.16–19.37	11.34	6.48–33.16
HPV53	14.06	5.37–29.65	12.55	9.64–28.81	10.12	7.30–21.30	8.98	5.19–17.68	11.38	6.14–24.91
HPV56	4.7	1.81–15.27	5.16	2.42–13.75	10.41	5.91–28.65	12.77	3.68–31.51	12.08	7.62–29.10
HPV68	10.3	8.14–22.34	11.44	9.36–33.13	8.73	4.67–19.63	9.61	7.0–-1.34	7.53	4.19–12.68
HPV51	-	-	-	-	2.15	1.06–8.22	3.61	0.39–8.12	2.74	1.02–6.34
HPV66	2.61	0.97-11.20	2.09	0.84-10.30	8.69	2.45–28.34	7.35	4.12–23.17	9.00	3.06–19.96
HPV6	-	-	-	-	3.11	0.36–26.83	2.46	0.34–24.21	2.94	1.04–14.01
HPV59	-	-	-	-	-	-	-	-	-	-
HPV31	5.74	2.34-12.43	5.97	1.14–11.87	5.53	1.64–14.13	8.73	2.66–13.41	7.37	3.10–17.01
HPV81	-	-	-	-	-	-	-	-	-	-
HPV45	-	-	-	-	2.61	0.54–8.37	-	-	-	-
HPV35	-	-	-	-	1.99	0.60-6.62	-	-	-	-
HPV39	-	-	-	-	-	-	-	-	-	-
HPV43	-	-	-	-	1.17	0.22–5.60	-	-	-	-
HPV83	-	-	-	-	2.34	1.04–5.24	-	-	-	-
HPV73	-	-	-	-	0.84	0.24–8.42	-	-	-	-
HPV82	-	-	-	-	-	-	-	-	-	-
HPV11	-	-	-	-	-	-	-	-	-	-
HPV42	-	-	-	-	-	-	-	-	-	-

**Table 3:** Age-specific prevalence of HPV.

**Age group (years old)**	**Single HPV +no. (%)**	**Multiple HPV+ no. (%)**	**HPV+ no. (%)**	**Total cases no.**
**<20**	22 (15.2%)	26 (17.9%)	48 (33.1%)	145
**21–30**	308 (15.3%)	210 (10.4%)	518 (25.7%)	2015
**31–40**	620 (19.6%)	155 (4.9%)	775 (24.5%)	3158
**41–50**	701 (17.9%)	137 (3.5%)	838 (21.4%)	3906
**51–60**	428 (16.9%)	76 (3.1%)	504 (20.1%)	2522
**>60**	137 (12.8%)	36 (3.3%)	173 (16.2%)	1070
**Total**	2216 (17.3%)	640 (5.0%)	2856 (22.3%)	12816

## Supplementary Materials

Supplementary Materials Table S1: HPV prevalence according to different cytological results.
