# Supplementary material for: Prevalence and Genotype Distribution of Human Papillomavirus in Invasive Cervical Cancer, Cervical Intraepithelial Neoplasia, and Asymptomatic Women in Southeast China
Source: Biomed Res Int. 2018 Oct 8;2018:2897937. doi: 10.1155/2018/2897937 (PMC6196990; doi:10.1155/2018/2897937)
Supplement: Supplementary Materials — Table S1: HPV prevalence according to different cytological results. [file 2897937.f1.docx]

TableS1. HPV prevalence according to different cytological results

| HPV  Infection | Normal | ASCUS | ASCH | LSIL | HSIL | CC |
| --- | --- | --- | --- | --- | --- | --- |
| HPVpositive | 574(5.8%) | 219(50.1 %) | 434(61.0%) | 167(86.0%) | 799(82.9%) | 663(100%) |
| Total | 9848 | 437 | 711 | 194 | 963 | 663 |
